# Editorial: Metabolic Intervention Based on Functional Biomaterial Strategy to Potentiate Cancer Immunotherapy, Volume I

**DOI:** 10.3389/fphar.2022.925673

**Published:** 2022-06-30

**Authors:** Bing Feng

**Affiliations:** ^1^ Institute of Bioengineering, École Polytechnique Fédérale de Lausanne (EPFL), Lausanne, Switzerland; ^2^ Institute of Materials Science and Engineering, EPFL, Lausanne, Switzerland

**Keywords:** metabolic intervention, antitumor immunity, targeting delivery, functional materials, cancer therapy

Tumor antigen specific CD8^+^ T cells are the main mediators of tumor destruction. Their intrinsic qualities-including differentiation, activation, proliferation, longevity, and effector function as well as durability-play important roles in determining the efficacy of immunotherapy (Li et al., 2019). These qualities are tightly and comprehensively regulated by metabolic activities. However, CD8^+^ T cells face metabolically hostile tumor environments and fierce competition for nutrients with tumor cells and other stromal cells (Chang et al., 2015). Thus, CD8^+^ T cells have to overcome metabolic disadvantages to mount a successful and sustained antitumor immune response.

Two possible directions have been taken towards enhancing the metabolic fitness of CD8^+^ T cells. One is to inhibit the metabolic activity of tumor cells to alleviate the nutrient-deprivation environment for CD8^+^ T cells in addition to killing tumor cells. Metabolic inhibition in tumor cells has been considered an innovative therapeutic approach as tumor cells characterized by high metabolic activities can quickly consume nutrients including glucose, amino acid as well as fatty acids. Most metabolic inhibitors are small molecules and cannot specifically modulate targets. Considering the metabolic similarities between tumor cells and CD8^+^ T cells, it is necessary to achieve tumor-specific metabolic inhibition without compromising the CD8^+^ T cells’ metabolism. Another approach, is to directly modulate the CD8^+^ T cells’ metabolic activities. However, it is still difficult to specifically modulate CD8^+^ T cells metabolism to enhance the T cells’ metabolic fitness. (DePeaux and Delgoffe, 2021).

In the last decade, functional biomaterials have been developed to target nanoparticles or make implantable scaffolds to achieve drug targeting delivery or regional treatment, to promote the targeting metabolic intervention therapy and modulate the tumor metabolic environment to potentiate cancer immunotherapy (Riley et al., 2019). This Research Topic aims to find feasible strategies for achieving specific metabolic interventions based on functional materials to improve the metabolic fitness of T cells. Contributors to the topic have proposed numerous intelligent strategies and findings in metabolism modulation or comprehensively reviewed recent progress in the area. Here, we briefly summarize this topic together with some thoughts on how to specifically modulate CD8 T cell metabolism.

**FIGURE 1:**
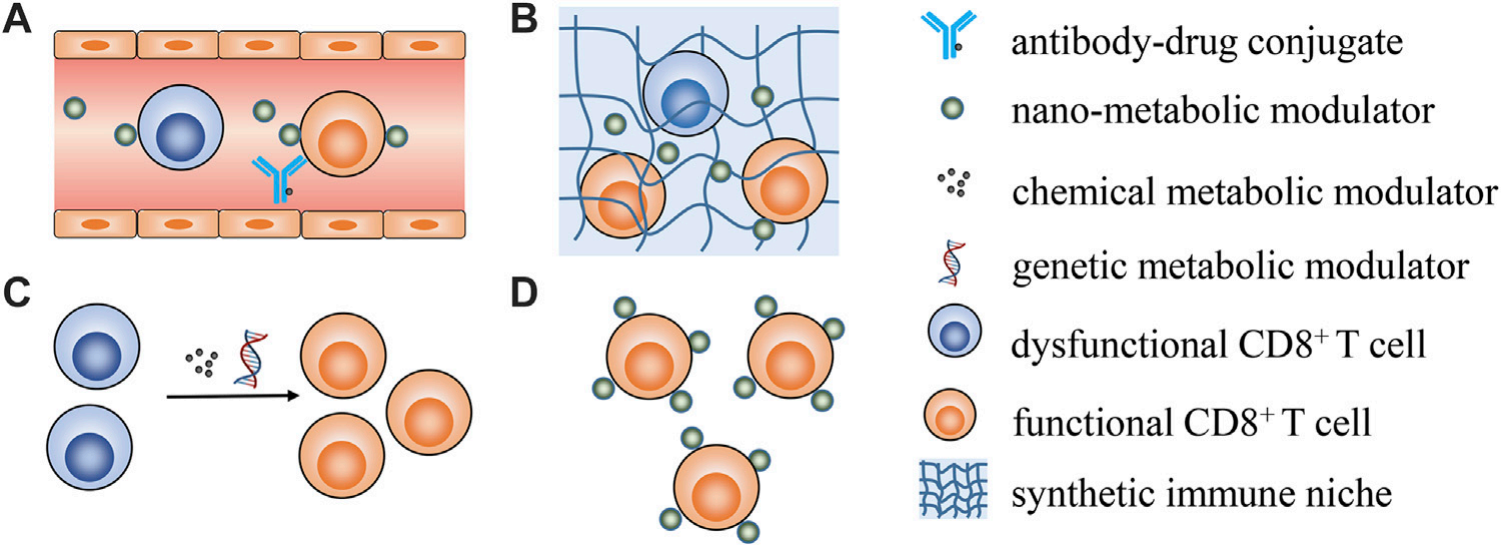
Possible strategies for metabolically manipulating CD8^+^ T cells to enhance cancer immunotherapy. **(A)** targeting peripheral CD8^+^ T cells utilizing antibody-drug conjugates or antibody-modified nanoparticles to specifically modulate CD8^+^ T cells metabolism; **(B)** employing synthetic immune niche to recruit CD8^+^ T cells and successive metabolic modulation; **(C)** leveraging genetic and pharmacological interventions to transiently modulate T cell metabolic properties *ex vivo* to generate metabolic fitness T cells for ACT; **(D)** functionalizing CD8^+^ T cells with nano-vehicles loading metabolic modulators *ex vivo* for real-time T cells metabolism modulation *in vivo* after ACT.

Zhao et al. reported that *lactobacillus* plantarum RS-09 could induce M1-type macrophage differentiation through the TLR2/NF‐κB signaling pathway and help to understand the mechanism by which RS-09 modulates the host immune response against pathogen infection. As M1 is reported to display tumor suppression effects and its differentiation is close to metabolism reprogramming, RS-09 is likely to be involved in metabolic interventions. Li et al. briefly summarize recent progress in using biogenic nanovehicles for RNA drug delivery and cancer therapy. Huang et al. provide an overview of the progress of deep eutectic solvents as active pharmaceutical ingredient delivery systems in the treatment of metabolic-related diseases. Tu et al. comprehensively summarize nanotechnology-based strategies for lipid metabolism regulation in the tumor microenvironment for enhanced anticancer immune responses, with insightful perspectives in this area. This review contributes to understanding of how to leverage nanotechnology to achieve lipid metabolism modulation and potentiate cancer immunotherapy. As no contributions have explored specifically modulating CD8^+^ T cells metabolism to date, some possible strategies are shared in Figure 1.

Most metabolic modulators are small molecules without T cell targeting properties, meaning how to specifically deliver metabolic modulators to CD8^+^ T cells will be a prerequisite for future studies. Antibody–drug conjugate is one promising choice for constructing CD8^+^ T cell specific antibody-metabolic modulators for CD8^+^ T cells metabolic modulation. Although the conception and clinical application of antibody–drug conjugates in targeting drug delivery has been confirmed, limited drug loading efficacy is one concern for antibody-drug conjugates (Van der Meel et al., 2019). The alternative choice is utilizing nano-formulations decorated with T cell specific antibodies as targeting vehicles (Cevaal et al., 2021). The feasibility of this approach has been confirmed by an elegant study where PLGA nanoparticles were modified with anti-CD8a F (ab’) (Chang et al., 2015) fragments that could specifically deliver TGFβ inhibitors to CD8^+^ T cells in the blood, lymphoid tissues, and tumors of mice (Schmid et al., 2017). The review by Tu et al. also introduces present progress in leveraging nanotechnology to directly target T cells and improve their fatty acid metabolism. Considering that the targeting efficacy will be compromised in solid tissues, this strategy may be mainly feasible in the treatment of haematological cancers to directly improve the metabolic fitness of peripheral circulating T cells. Synthetic immune niches have been quickly advanced in cancer immunotherapy (Weiden et al., 2018). Engineered three-dimensional scaffolds could be used as an immune niche to locally deliver cancer vaccines or even CD8^+^ T cells (Dellacherie et al., 2019). Moreover, antigen specific CAR-T cells could be rapidly generated *in vivo* using an implantable scaffold, which could first recruit peripheral T cells and genetically engineer them into CAR-T cells for cancer treatment (Agarwalla et al., 2022). Thus, leveraging a similar strategy for *in vivo* metabolic manipulation of T cells within an injectable hydrogel or implantable scaffold should be feasible.

In contrast to *in vivo* T cell metabolic modulation, *ex vivo* manipulation is much easier and more flexible (Kishton et al., 2017). The metabolic status of T cells could be sophisticatedly regulated to control T cell differentiation and effector function using various chemical reagents or genetic engineering methods for adoptive cell transfer (ACT) treatment (Gattinoni et al., 2009). However, transient metabolic modulation cannot last when T cells are transferred into the body, as they gradually become exhausted and dysfunctional. Functionalizing the T cells with nanoparticle loading metabolic modulators *ex vivo* is a smart strategy for real-time T cell metabolism modulation after ACT. It has been reported that CD8^+^ T cells anchored with liposomal avasimibe could effectively modulate T cell cholesterol metabolism and specifically increase the cholesterol content in the T cell membrane for sustained T cell activation (Hao et al., 2020).

T cell metabolic activity is closely associated with effective antitumor immunotherapy. Metabolic improvement of CD8^+^ T cells, utilizing different feasible strategies including genetic and pharmacological interventions based on functional materials have the potential to enhance cancer immunotherapy.
